# Comparing Entrustment Decision-Making Outcomes of the Core Entrustable Professional Activities Pilot, 2019-2020

**DOI:** 10.1001/jamanetworkopen.2022.33342

**Published:** 2022-09-26

**Authors:** David R. Brown, Jeremy J. Moeller, Douglas Grbic, Dorothy A. Andriole, William B. Cutrer, Vivian T. Obeso, Mark D. Hormann, Jonathan M. Amiel

**Affiliations:** 1 Division of Family and Community Medicine, Department of Humanities, Health, and Society, Florida International University Herbert Wertheim College of Medicine, Miami; 2 Department of Neurology, Yale University School of Medicine, New Haven, Connecticut; 3 Medical Education Research, Association of American Medical Colleges, Washington, District of Columbia; 4 Department of Pediatrics, Division of Critical Care Medicine at Vanderbilt University School of Medicine, Nashville, Tennessee; 5 Division of Internal Medicine, Department of Translational Medicine, Florida International University Herbert Wertheim College of Medicine, Miami; 6 Division of Community and General Pediatrics, Department of Pediatrics, McGovern Medical School at UTHealth, Houston, Texas; 7 Dean’s Office, Columbia University Vagelos College of Physicians and Surgeons, New York, New York; 8 Department of Psychiatry, Columbia University Vagelos College of Physicians and Surgeons, New York, New York

## Abstract

**Importance:**

Gaps in readiness for indirect supervision have been identified for essential responsibilities encountered early in residency, presenting risks to patient safety. Core Entrustable Professional Activities (EPAs) for entering residency have been proposed as a framework to address these gaps and strengthen the transition from medical school to residency.

**Objective:**

To assess progress in developing an entrustment process in the Core EPAs framework.

**Design, Setting, and Participants:**

In this quality improvement study in the Core EPAs for Entering Residency Pilot, trained faculty made theoretical entrustment determinations and recorded the number of workplace-based assessments (WBAs) available for each determination in 2019 and 2020. Four participating schools attempted entrustment decision-making for all graduating students or a randomly selected subset of students. Deidentified, individual-level data were merged into a multischool database.

**Interventions:**

Schools implemented EPA-related curriculum, WBAs, and faculty development; developed systems to compile and display data; and convened groups to make theoretical summative entrustment determinations.

**Main Outcomes and Measures:**

On an EPA-specific basis, the percentage of students for whom an entrustment determination could be made, the percentage of students ready for indirect supervision, and the volume of WBAs available were recorded.

**Results:**

Four participating schools made 4525 EPA-specific readiness determinations (2296 determinations in 2019 and 2229 determinations in 2020) for 732 graduating students (349 students in 2019 and 383 students in 2020). Across all EPAs, the proportion of determinations of “ready for indirect supervision” increased from 2019 to 2020 (997 determinations [43.4%] vs 1340 determinations [60.1%]; 16.7 percentage point increase; 95% CI, 13.8-19.6 percentage points; *P* < .001), as did the proportion of determinations for which there were 4 or more WBAs (456 of 2295 determinations with WBA data [19.9%] vs 938 [42.1%]; 22.2 percentage point increase; 95% CI, 19.6-24.8 percentage points; *P* < .001). The proportion of EPA-specific data sets considered for which an entrustment determination could be made increased from 1731 determinations (75.4%) in 2019 to 2010 determinations (90.2%) in 2020 (14.8 percentage point increase; 95% CI, 12.6-16.9 percentage points; *P* < .001). On an EPA-specific basis, there were 5 EPAs (EPA 4 [orders], EPA 8 [handovers], EPA 10 [urgent care], EPA 11 [informed consent], and EPA 13 [patient safety]) for which few students were deemed ready for indirect supervision and for which there were few WBAs available per student in either year. For example, for EPA 13, 0 of 125 students were deemed ready in 2019 and 0 of 127 students were deemed ready in 2020, while 0 determinations in either year included 4 or more WBAs.

**Conclusions and Relevance:**

These findings suggest that there was progress in WBA data collected, the extent to which entrustment determinations could be made, and proportions of entrustment determinations reported as ready for indirect supervision. However, important gaps remained, particularly for a subset of Core EPAs.

## Key Points

**Question:**

What was learned about entrustment decision-making over 2 graduating cohorts of the Core Entrustable Professional Activities (EPAs) Pilot from 2019 to 2020?

**Findings:**

In this quality improvement study among 732 graduating medical students, “ready for indirect supervision” determinations increased significantly from 2019 to 2020 (43.4% vs 60.1%) across all EPA-specific determinations combined, as did the availability of workplace-based assessments (WBAs). There were 5 EPAs (orders, handovers, urgent care, informed consent, and patient safety) for which few students were deemed ready and few WBAs were available in either year.

**Meaning:**

These findings suggest that pilot schools made progress in building programs of assessment, yet important gaps remain on the path to entrustment for entering residency.

## Teaser

This quality improvement study assesses progress from 2019 to 2020 in a Core Entrustable Professional Activities entrustment decision-making program for graduating medical students entering residency.

## Introduction

### Problem Description

Gaps in readiness for indirect supervision upon transition to residency have been identified for important resident responsibilities.^1,2^ Entrustable Professional Activities (EPAs)^3^ have been proposed as a framework to advance competency-based medical education (CBME),^3^ focusing assessment on observable day-to-day professional activities^4^ and the level of supervision required for each learner.

### Available Knowledge

CBME is increasing in influence as a conceptual model, while implementation remains challenging.^5,6,7,8,9^ Graduate medical education (GME) has led implementation of CBME,^5,10^ milestones,^11^ programmatic assessment,^12,13,14^ clinical competency committee processes,^15,16,17^ and EPAs.^9,18,19^ The Accreditation Council for Graduate Medical Education requires reporting of milestones, and early assessments of validity and reliability of milestones ratings are underway.^6,11^ Lack of a uniform undergraduate medical education (UME) process for evaluating clinical competency, coupled with concerns about competency gaps for graduating students, focused attention on CBME in UME.^20,21^

### Rationale

Aiming to address these gaps and to advance CBME in UME, the Association of American Medical Colleges (AAMC) convened a panel to draft a set of Core EPAs for Entering Residency^21,22^ (Table 1). In a survey of internal medicine program directors, most respondents indicated that graduating students “must” or “should” possess skills to perform most Core EPAs without direct supervision.^23^ An Association of Program Directors in Surgery statement concludes, “Students should achieve entrustability” in Core EPAs.^24^ Feasibility of small-scale (approximately 4 students per year at each of 4 schools), time-variable advancement from UME to GME based in part on Core EPAs has been demonstrated in the Education in Pediatrics Across the Continuum initiative.^25,26^

**Table 1.** Entrustment Decision-Making Outcomes^a^

EPA	Students with data, No.	Total students, No. (%)	Entrustment determinations for which a determination of readiness could be made^b^			
			No./No. (%)		Difference (95% CI), percentage points	2-Sided *P* value
			2019	2020		
1: Gather a history and perform a physical examination	732	732 (100.0)	291/349 (83.4)	377/383 (98.4)	15.1 (11.0 to 19.2)	<.001
2: Prioritize a differential diagnosis following a clinical encounter	182	182 (24.9)	70/100 (70.0)	77/82 (93.9)	23.9 (13.5 to 34.3)	<.001
3: Recommend and interpret common diagnostic and screening tests	182	182 (24.9)	69/100 (69.0)	77/82 (93.9)	24.9 (14.5 to 35.3)	<.001
4: Enter and discuss orders and prescriptions	182	182 (24.9)	79/100 (79.0)	74/82 (90.2)	11.2 (0.0 to 21.5)	.04
5: Document a clinical encounter in the patient record	428	428 (58.5)	182/204 (89.2)	214/224 (95.5)	6.3 (1.3 to 11.4)	.01
6: Provide an oral presentation of a clinical encounter	662	662 (90.4)	286/324 (88.3)	333/338 (98.5)	10.2 (6.5 to 14.0)	<.001
7: Form clinical questions and retrieve evidence to advance patient care	416	416 (56.8)	193/220 (87.7)	195/196 (99.5)	11.8 (7.3 to 16.2)	<.001
8: Give or receive a patient handover to transition care responsibility	252	252 (34.4)	93/125 (74.4)	99/127 (78.0)	3.6 (−7.0 to 14.1)	.51
9: Collaborate as a member of an interprofessional team	416	416 (56.8)	180/220 (81.8)	180/196 (91.8)	10.0 (3.6 to 16.4)	.003
10: Recognize a patient requiring urgent/emergent care and initiate evaluation/management	182	182 (24.9)	72/100 (72.0)	72/82 (87.8)	15.8 (4.5 to 27.1)	.009
11: Obtain informed consent for tests/procedures	182	182 (24.9)	62/100 (62.0)	66/82 (80.5)	18.5 (5.7 to 31.3)	.007
12: Perform general procedures of a physician	457	457 (62.4)	129/229 (56.3)	142/228 (62.3)	5.9 (−3.0 to 14.9)	.20
13: Identify system failures and contribute to a culture of safety and improvement	252	252 (34.4)	25/125 (20.0)	104/127 (81.9)	61.9 (52.2 to 71.6)	<.001
Total	NA	NA	1731/2296 (75.4)	2010/2229 (90.2)	14.8 (12.6 to 16.9)	<.001

Abbreviations: EPA, entrustable professional activity; NA, not applicable.

^a^ Among 4 schools combined, including schools that made entrustment determinations for the listed EPA in both 2019 (349 students) and 2020 (383 students).

^b^ The percentage is the number of students who were ready plus those progressing plus those not progressing divided by the number of students with data for the EPA.

In 2014, the AAMC convened 10 US medical schools to explore feasibility of implementing the Core EPA framework, including summative entrustment decision-making, for entire classes of students. Pilot schools established steering committees; concept groups for curriculum and assessment,^27^ faculty development,^28^ and entrustment^29^; and 13 EPA-specific workgroups. Early pilot work focused on developing tools for curriculum and assessment,^30,31^ fostering faculty development,^32^ and establishing entrustment committee structures modeled on the GME clinical competency committee.^33^

Trained faculty were convened to make determinations about readiness for indirect supervision looking at multimodal performance data, including Workplace Based Assessments (WBAs) with entrustment-supervision scales.^34,35^ These determinations were generally completed within a day after direct observation in the workplace.^36,37^

### Specific Aims

Lomis et al^38^ described a principal aim of the pilot: to develop theoretical summative determinations of each student’s readiness to perform 13 Core EPAs in 2019 “to inform the feasibility of implementing the Core EPAs construct in UME programs.” In an evaluation of decision-making for the 2019 graduating cohort, fewer than half of EPA-specific entrustment determinations had a finding of “ready for indirect supervision,” and more than one-quarter had a report of “could not make an entrustment determination.”^39^ This study aimed to evaluate what further progress was made with the 2020 graduating cohort in implementing programmatic assessment^14,40,41^ and establishing a summative entrustment process using AAMC Core EPAs.

## Methods

This quality improvement study was reported using the Standards for Quality Improvement Reporting Excellence (SQUIRE) 2.0 reporting guidelines. Human Research Protection Program staff at the AAMC determined that this study was exempt from further institutional review board (IRB) review and informed consent because it did not constitute human participants research as defined in 45 CFR §46 given that the AAMC access only deidentified data. At 2 of 4 participating institutions (Columbia University Vagelos College of Physicians and Surgeons and Vanderbilt University School of Medicine), the study was submitted for IRB review as a new study; at the other 2 participating institutions (Florida International University Herbert Wertheim College of Medicine and McGovern Medical School at UTHealth Houston), the study was submitted for IRB review as a modification of a preexisting, IRB-reviewed protocol for the entire Core EPAs pilot study. At all participating institutions, this study was deemed exempt from further IRB review and informed consent as defined in 45 CFR §46.

### Initial Intervention Steps and Their Evolution Over Time

The AAMC and each institution (through letters from the dean of the medical school and the curriculum committee) committed to a 5-year pilot, initiated in July 2014. The AAMC and all 10 institutions subsequently agreed to a 2-year extension.

The first year served as a planning phase, in which participating institutions developed as a community, established working groups, developed guiding principles, and planned curricula, assessment strategies, faculty development, and pathways to entrustment.^38^ Each school agreed to pilot a minimum of 4 EPAs.

With the entering class of 2015, institutions endeavored to initiate curricula, assessments, and faculty development. Each institution developed plans to render nonbinding entrustment determinations for graduating students. Schools that initiated implementation for the entering class of 2015 developed plans for trained faculty groups to make theoretical readiness determinations starting with the 2019 graduating class.

The entrustment workgroup of the pilot used an iterative process of discussions, data collection, and reflection to describe principles, plans, and activities related to the entrustment process^29^; choices schools made about the entrustment process, why choices were made, and challenges with the entrustment process^33^; data considered for each EPA; and results of entrustment decision-making for the first graduating cohort.^39^

Due to local circumstances (eg, curriculum overhaul or Liaison Committee for Medical Education site visit), some schools elected to extend planning and begin implementation with the entering class of 2016 or 2017 (corresponding to the 2020 or 2021 graduating class). A decision to extend the pilot was made in 2018. An initial round of entrustment determinations and compilation of data was completed in 2019 (for the graduating class of 2019), and a second round was completed in 2020 (for the graduating class of 2020).

### Context

Implementing a centralized entrustment process at a medical school involves a major curricular change and poses a variety of cultural, logistical, analytic, psychometric, and ethical challenges.^33^ Evaluation of entrustment decision-making for the 2019 graduating cohort highlighted multifactorial challenges in assessment of some of these activities in the workplace,^39^ suggesting the need to consider curriculum content revisions and increase availability of WBAs and other assessment data.

### Interventions

Schools implemented various improvements based on this first cohort of data collection. These changes included increasing the number of required WBAs for some EPAs, increasing the number of end-of-rotation assessments mapped to EPAs, enhancing curriculum, enhancing data visualization for the entrustment process, providing additional faculty development, and using alternative methods, such as simulation to assess skills. For the study of interventions, 4 schools piloted entrustment decision-making and shared deidentified data for some or all graduating students in both 2019 and 2020.

### Measures

The entrustment workgroup, AAMC staff, and Core EPA team leaders collaborated to create a data set for evaluation of entrustment decision-making outcomes across sites. These groups jointly determined that individual-level, deidentified data would be shared for multischool analysis.

Among the uniform set of items at the individual level collected for each EPA-specific instance of entrustment decision-making were readiness determinations (1 of 4 choices: *ready* for indirect supervision, *progressing* but not yet ready for indirect supervision, *not progressing* toward readiness for indirect supervision, or *could not make a determination*) and number of WBAs available for that determination (choices of 0, 1-3, 4-10, 11-15, and >15). Based on preliminary analysis of volumes of WBAs available, we created a dichotomous variable for WBAs available (0-3 vs ≥4). Schools also incorporated other available assessments into the entrustment process, as described previously.^33,39^

### Statistical Analysis

Data were analyzed for EPAs that each participating school considered in both 2019 and 2020. Each school considered all students or a similarly sized, randomly selected subset in both years. Proportional representation from each school was roughly similar by year. We compiled descriptive statistics and assessed between-year differences in percentages using 2-sample test of proportions, and we assessed associations between entrustment determinations and number of WBAs using χ^2^, with a 2-sided *P* < .05 considered significant. All analyses were performed using Stata statistical software version 17 (StataCorp).

## Results

### Details of the Process Measures and Outcome

Among 4 participating schools, for EPAs that a given school considered in both years (minimum of 4 EPAs per school), the schools made 4525 EPA-specific entrustment determinations (2296 determinations in 2019 and 2229 determinations in 2020) for 732 students (349 students in 2019 and 383 students in 2020) (Table 1). Proportions of students with data for each EPA ranged from 25% to 100% (182 students [24.9%] for EPAs 2, 3, 4, 10, and 11 to 732 students for EPA 1 [100%]). The proportion of EPA-specific data sets considered for which an entrustment determination could be made (1 of 3 choices, including not progressing towards readiness, progressing towards readiness, and ready for indirect supervision and not including could not make a determination) increased from 1731 determinations in 2019 (75.4%) to 2010 determinations in 2020 (90.2%) (14.8 percentage point increase; 95% CI, 12.6-16.9 percentage points; *P* < .001). This proportion varied on an EPA-specific basis, from 25 of 125 EPA 13 (safety) determinations (20.0%) to 182 of 204 (EPA 5) (notes) determinations (89.2%) in 2019 and from 142 of 228 EPA 12 (procedures) determinations (62.3%) to 195 of 196 EPA 7 (evidence) determinations (99.5%) in 2020. The proportion increased from 2019 to 2020 for every EPA examined (Table 1) except EPA 8 (handovers; 3.6 percentage point increase; 95% CI, –7.0 to 14.1; *P* = .51) and EPA 12 (procedures; 5.9 percentage point increase; 95% CI, –3.0 to 14.9; *P* = .20). Additionally, the proportion of EPA-specific data sets for which a determination of ready for indirect supervision was made increased from 997 determinations in 2019 (43.4%) to 1340 determinations in 2020 (60.1%; 16.7 percentage point increase; 95% CI, 13.8 to 19.6; *P* < .001).

As shown in the Figure, the change in distribution from 2019 to 2020 of types of entrustment determinations for 3 choices of not progressing towards readiness, progressing towards readiness, and ready for indirect supervision varied on an EPA-specific basis, with data for could not make a determination excluded. For EPAs 1, 2, 3, 6, 7, and 12, there was a significant change in the proportion of determinations that were ready for indirect supervision in 2020 vs 2019, ranging from differences of −11.1 percentage points (95% CI, −19.1% to −3.2%; *P* = .007) for EPA 7 to 43.4 percentage points (95% CI, 28.8 to 58.1 percentage points; *P* < .001) for EPA 3 (eTable in Supplement 1).

**Figure.**
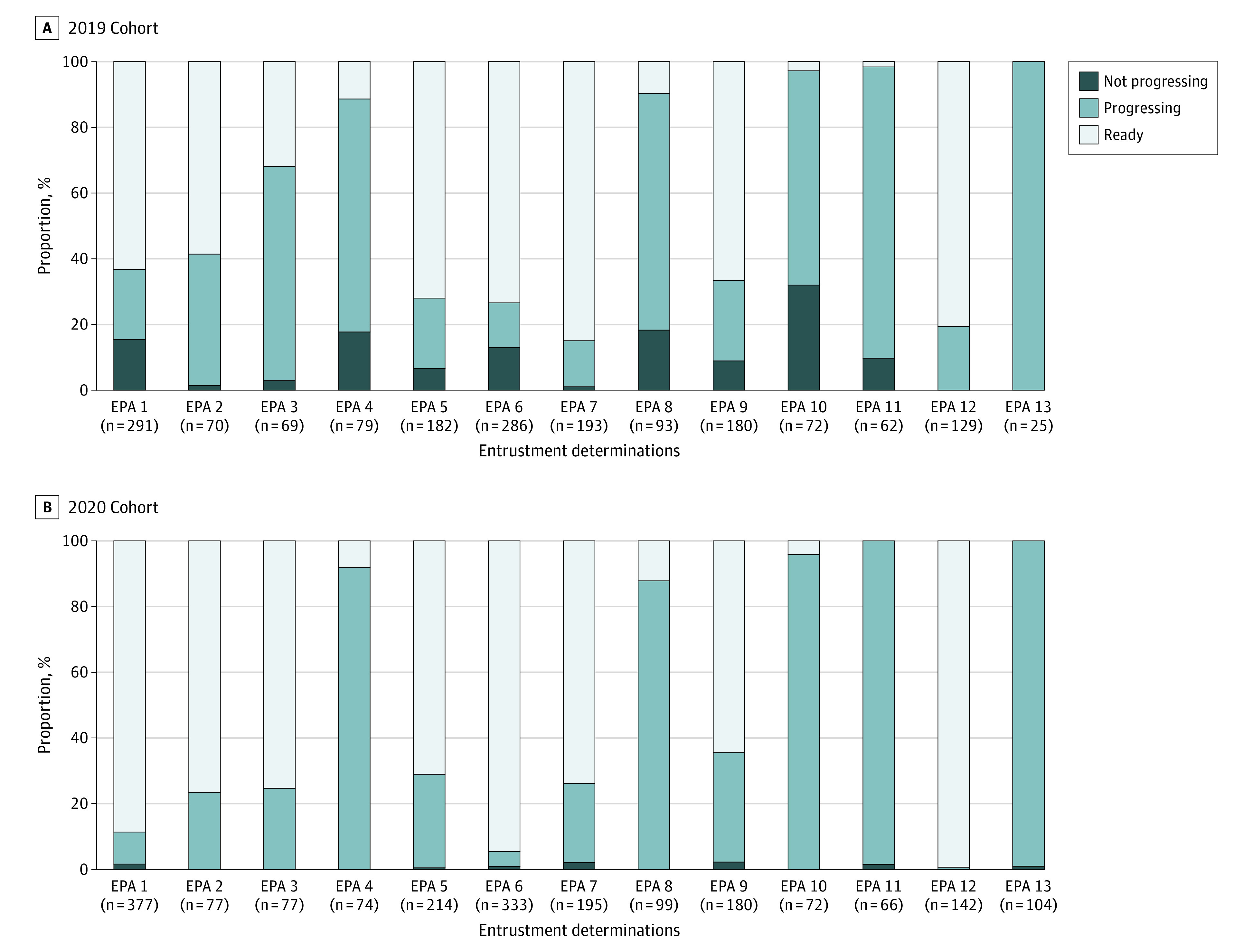
Distribution of Entrustment Determinations The distribution of types of entrustment determinations are presented only among students for whom determinations were made for 3 choices of “not progressing towards readiness,” “progressing towards readiness,” and “ready for indirect supervision,” with “could not make a determination” data excluded. See eTable in Supplement 1 for Entrustable Professional Activity (EPA)–specific changes in 2020 vs 2019 in proportions deemed ready. The change in the proportion ready for indirect supervision was significant for EPAs 1, 2, 3, 6, 7, and 12.

As shown in Table 2, the proportion of EPA-specific data sets that included 4 or more WBAs increased from 456 of 2295 determinations with WBA data in 2019 (19.9%) to 938 determinations (42.1%) in 2020 (22.2 percentage point increase; 95% CI, 19.6-24.8 percentage points; *P* < .001). This proportion varied on an EPA-specific basis, from, for example, 0 of 125 determinations for EPA 13 to 66 of 100 determinations (66.0%) for EPA 2 in 2019 and from, for example, 0 of 127 determinations (0%) for EPA 13 to 75 of 82 determinations (91.5%) for EPA 2 in 2020. EPA-specific proportions increased from 2019 to 2020 for EPAs 1 through 3, 5 through 7, 9, and 12 but not for EPAs 4, 8, or 10 or for EPAs 11 and 13 (0% availability in both years).

**Table 2.** Availability of ≥4 WBAs

EPA	4 or More WBAs available			
	Determinations, No./Total No. (%)		Difference (95% CI), percentage points	2-sided *P* value
	2019^a^	2020		
Total	456/2295 (19.9)	938/2229 (42.1)	22.2 (19.6 to 24.8)	<.001
1	88/349 (25.2)	254/383 (66.3)	41.1 (34.5 to 47.7)	<.001
2	66/100 (66.0)	75/82 (91.5)	25.5 (14.4 to 36.5)	<.001
3	2/100 (2.0)	71/82 (86.6)	84.6 (76.7 to 92.5)	<.001
4	8/100 (8.0)	6/82 (7.3)	−0.7 (−8.4 to 7.1)	.86
5	6/203a (3.0)	25/224 (11.2)	8.2 (3.5 to 12.9)	.001
6	99/324 (30.6)	209/338 (61.8)	31.3 (24.1 to 38.5)	<.001
7	77/220 (35.0)	151/196 (77.0)	42 (33.4 to 50.7)	<.001
8	2/125 (1.6)	6/127 (4.7)	3.1 (−1.2 to 7.4)	.16
9	104/220 (47.3)	117/196 (59.7)	12.4 (2.9 to 21.9)	.01
10	4/100 (4.0)	4/82 (4.9)	0.9 (−5.2 to 6.9)	.77
11	0/100	0/82	0 (NA)	NA
12	0/229	20/228 (8.8)	8.8 (5.1 to 12.4)	<.001
13	0/125	0/127	0 (NA)	NA

Abbreviations: EPA, entrustable professional activity; NA, not applicable; WBA, workplace-based assessment.

^a^ WBA data missing for 1 student for EPA 5 due to an oversight.

Proportions of students with determinations that they were ready for indirect supervision in 2020 was highest for EPAs 1 (334 of 383 students [87.2%]) and 6 (315 of 338 students [93.2%]). They were intermediate for EPAs 2, 3, 5, 7, 9, and 12 (ranging from 116 of 196 students [59.2%] for EPA 9 to 144 of 196 students [73.5%] for EPA 7), and lowest for EPAs 4, 8, 10, 11, and 13 (<10%; for example, 3 of 82 students [3.7%] for EPA 4 and 0 of 127 students for EPA 13) (Table 3). Proportions with determinations that they were ready for indirect supervision for 2019 ranged from, for example, 0 of 125 students for EPA 13 to 164 of 220 (74.5%) students for EPA 7 (Table 3).

**Table 3.** Percentage of Students Determined as Ready for Indirect Supervision by EPA and RIME Category

RIME category	EPA	Students, No./total No. (%) (N = 732)^a^	
		2019 (n = 349)	2020 (n = 383)
Reporter	1	184/349 (52.7)	334/383 (87.2)
	5	131/204 (64.2)	152/224 (67.9)
	6	210/324 (64.8)	315/338 (93.2)
Interpreter	2	41/100 (41.0)	59/82 (72.0)
Interpreter and manager	3	22/100 (22.0)	58/82 (70.7)
	10	2/100 (2.0)	3/82 (3.7)
Manager	4	9/100 (9.0)	6/82 (7.3)
	8	9/125 (7.2)	12/127 (9.4)
	11	1/100 (1.0)	0/82
	12	104/229 (45.4)	141/228 (61.8)
Educator	7	164/220 (74.5)	144/196 (73.5)
	9	120/220 (54.6)	116/196 (59.2)
	13	0/125 (20.0)	0/127

Abbreviations: EPA, entrustable professional activity; RIME, reporter interpreter manager educator.

^a^ Total No. varies because not all students had data for each EPA.

### Contextual Elements That Interacted With Interventions

The creation of toolkits including key functions, associated competencies, and behavioral expectations for each EPA helped to develop a shared mental model across schools.^30^ The pilot engaged in a “goldfish bowl” training exercise for level-setting prior to starting to make formal entrustment determinations. The formative nature of the pilot was associated with the robustness of implementation. Entrustment determinations did not have high-stakes summative implications. School-specific differences regarding final-year rotation requirements could also have been associated with these outcomes.^42^ Suspension of face-to-face clinical activities due to the COVID-19 pandemic in 2020 may have been associated with the numbers of WBAs for some EPAs. Entrustment committees met face to face in 2019 and virtually in 2020, which may also have been associated with outcomes.^16^ Experiences working as a team in 2019 may have been associated with reduced challenges in working virtually to some extent. Teams were more experienced in the process in 2020 than in 2019. Faculty also may have recalibrated as they became more used to the process, returning to a “stance of presuming readiness.”^7^

### Observed Associations of Interventions and Relevant Contextual Elements with Outcomes

As shown in Table 4, distribution of entrustment determinations by WBA availability varied across EPAs. The presence of 4 or more WBAs (vs 0-3 WBAs) was generally associated with higher proportions of ready and progressing determinations and lower proportions of not progressing determinations or could not make determination outcomes. For example, for 141 determinations with 4 or more WBAs vs 41 determinations with 0 to 3 WBAs in EPA 2, there were 97 determinations (68.8%) vs 3 determinations (7.3%) that were ready, 43 determinations (30.5%) vs 3 determinations that were progressing, 1 determination (0.7%) vs 0 determinations that were not progressing, and 0 determinations vs 35 determinations (85.4%) that could not be made (P < .001) (Table 4). However*,* this was not so for every EPA. For example, for EPAs 1, 5, and 6, ready for indirect supervision determinations were made for approximately two-thirds of students whose EPA-specific data sets included 0 to 3 WBAs (eg, 250 of 390 students for EPA 1 [64.1%]), suggesting that availability and quality of additional assessment data to inform entrustment committee determinations varied across EPAs.^43^

**Table 4.** Entrustment Determination by WBA Availability (2019 and 2020 Combined)

WBAs, No.	Determination, No. (%)^a^				Total determinations, No.	2-sided *P* value^b^
	Could not make determination	Ready for indirect supervision	Progressing toward readiness	Not progressing toward readiness		
EPA 1						
Total	64 (8.7)	518 (70.8)	99 (13.5)	51 (7.0)	732	<.001
0-3	62 (15.9)	250 (64.1)	37 (9.5)	41 (10.5)	390	
≥4	2 (0.6)	268 (78.4)	62 (18.1)	10 (2.9)	342	
EPA 2						
Total	35 (19.2)	100 (55.0)	46 (25.3)	1 (0.7)	182	<.001
0-3	35 (85.4)	3 (7.3)	3 (7.3)	0	41	
≥4	0	97 (68.8)	43 (30.5)	1 (0.6)	141	
EPA 3						
Total	36 (19.8)	80 (44.0)	64 (35.2)	2 (1.1)	182	<.001
0-3	36 (33.0)	23 (21.1)	48 (44.0)	2 (1.8)	109	
≥4	0	57 (78.1)	16 (21.9)	0	73	
EPA 4						
Total	29 (15.9)	15 (8.2)	124 (68.1)	14 (7.7)	182	<.001
0-3	29 (17.3)	1 (0.6)	124 (73.8)	14 (8.3)	168	
≥4	0	14 (100.0)	0 (0)	0 (0)	14	
EPA 5						
Total	32 (7.5)	283 (66.3)	99 (23.2)	13 (3.0)	427	.001
0-3	32 (8.1)	252 (63.6)	99 (25.0)	13 (3.3)	396	
≥4	0	31 (100.0)	0	0	31	
EPA 6						
Total	43 (6.5)	525 (79.3)	54 (8.2)	40 (6.0)	662	<.001
0-3	43 (12.2)	246 (69.5)	28 (7.9)	37 (10.5)	354	
≥4	0	279 (90.6)	26 (8.4)	3 (1.0)	308	
EPA 7						
Total	28 (6.7)	308 (74.0)	74 (17.8)	6 (1.4)	416	<.001
0-3	28 (14.9)	117 (62.2)	41 (21.8)	2 (1.1)	188	
≥4	0	191 (83.8)	33 (14.5)	4 (1.8)	228	
EPA 8						
Total	60 (23.8)	21 (8.3)	154 (61.1)	17 (6.8)	252	<.001
0-3	60 (24.6)	13 (5.3)	154 (63.1)	17 (7.0)	244	
≥4	0	8 (100.0)	0	0	8	
EPA 9						
Total	56 (13.5)	236 (56.7)	104 (25.0)	20 (4.8)	416	<.001
0-3	52 (26.7)	30 (15.4)	102 (52.3)	11 (5.6)	195	
≥4	4 (1.8)	206 (93.2)	2 (0.9)	9 (4.1)	221	
EPA 10						
Total	38 (20.9)	5 (2.8)	116 (63.7)	23 (12.6)	182	<.001
0-3	38 (21.8)	0	113 (64.9)	23 (13.2)	174	
≥4	0	5 (62.5)	3 (37.5)	0	8	
EPA 11						
Total	54 (29.7)	1 (0.6)	120 (65.9)	7 (3.9)	182	NA^c^
0-3	54 (29.7)	1 (0.6)	120 (65.9)	7 (3.9)	182	
≥4	0	0	0	0	0	
EPA 12						
Total	186 (40.7)	245 (53.6)	26 (5.7)	0	457	<.001
0-3	186 (42.6)	226 (51.7)	25 (5.7)	0	437	
≥4	0	19 (95.0)	1 (5.0)	0	20	
EPA 13						
Total	123 (48.8)	0	128 (50.8)	1 (0.4)	252	NA^c^
0-3	123 (48.8)	0	128 (50.8)	1 (0.4)	252	
≥4	0	0	0	0	0	

Abbreviations: EPA, entrustable professional activity; NA, not applicable; WBA, workplace-based assessment.

^a^ Percentages are out of the total number in the row and may not total exactly 100 due to rounding.

^b^ χ^2^ test of association.

^c^ No students with 4 or more WBAs for this EPA.

### Unintended Consequences

Entrustment under the Core EPA Pilot guiding principles was a complex intervention. It shed light on systems required for programmatic assessment and data compilation and highlighted a lack of workplace roles for students for several key clinical tasks.

### Details About Missing Data

Due to varying time frames for implementation, 2 schools provided entrustment data to the AAMC for graduating students in 2020 only (and so were not eligible to be included in this study). Due to local differences in implementation, data compilation issues, and disruptions related to the COVID-19 pandemic, the remaining 4 schools did not generate data for the multi-institutional data set in either 2019 or 2020. For schools that implemented more than the minimum of 4 EPAs, we excluded EPA-specific data that pertained to a given EPA implemented at a school and considered by its entrustment committee in only 1 of these years.

## Discussion

### Summary

In this quality improvement study, the proportions of decision-making instances for which a determination could be made, that were ready for indirect supervision, and that were informed by 4 or more WBAs increased in 2020 compared with 2019 overall and also on an EPA-specific basis for EPAs 1 to 3 and 6. We also observed increases in 2 of these 3 measures for EPAs 5, 7, 9, and 12. Together, these increases suggest progress in implementation of a program of assessment using the Core EPAs framework. After reviewing first cohort data, schools increased the number of WBAs required and found additional places for curriculum and assessments, which was associated with more available data for entrustment committees to consider for the second cohort. Schools also worked to improve display of data and training for entrustment committees. However, in contrast to progress made for 8 of 13 EPAs, determinations for the remaining 5 EPAs (EPAs 4, 8, 10, 11, and 13) remained challenging given that WBA availability did not increase and fewer than 10% of students considered for each of these EPAs were determined to be ready for indirect supervision in either year.

### Interpretation

The highest proportions of readiness for indirect supervision was observed among a subgroup of EPAs broadly taught and assessed throughout the UME curricula, including EPAs 1 to 3, 5 to 7, and 9. Meyer et al^44^ contextualized Core EPAs within the Reporter-Interpreter-Manager-Educator (RIME) framework. EPAs 1, 2, 3, 5, and 6 align with the reporter or interpreter level. Colbert-Getz et al^43^ found that EPAs 1, 2, 5, 6, and 9 were commonly addressed in clerkship narrative assessments. With process improvement put in place after the 2019 cohort of entrustment decision-making, we observed in 2020 that there were WBAs available for these EPAs for the most part and most students for whom entrustment decision-making was undertaken in these EPAs were determined to be ready to perform these activities with indirect supervision. Relatively high proportions of students in our study for whom entrustment decision-making was undertaken in EPAs 7 and 9 were determined to be ready for indirect supervision for these 2 EPAs. Meyer et al^44^ suggested that these EPAs aligned with the high-level educator role; however, pilot schools and others^43^ found that these EPAs were “well represented in the UME experience.”^45^

EPAs 4 and 8 may align better with the senior level subinternship curriculum.^42,43,45^ However, even at this level, opportunities to perform these EPAs may be limited at participating schools or supervision may be “not sufficiently intentional to collect evidence robust enough to substantiate entrustment decisions.”^45^

EPAs 10, 11, and 13 involve high-level skills (ie, manager or educator in the RIME framework). Our findings suggest that for these skills, current clinical environments may not provide students the opportunity for meaningful workplace participation or assessment.^33,43,45^

EPA 10 (urgent care) is commonly taught through didactics and simulation, but meaningful opportunities for students to demonstrate and be assessed on skills in the clinical environment prior to graduation may be quite limited. EPA 12 includes several procedural skills (eg, intravenous line, bladder catheterization, cardiopulmonary resuscitation, and bag-mask ventilation)^30^ for which the role of physicians and residents, nurses, and other health professionals varies by specialty and location; there were few WBAs available in this study; and simulation is commonly used in teaching and assessment.

### Limitations

This study has several limitations. The primary data for the main outcome measure came from 4 schools and 2 cohorts of a formative pilot of Core EPAs implementation. Furthermore, except for EPA 1 (for which we had data from all students in our data set), we had EPA-specific data for various subsets of students in the entire data set because of school differences in particular EPAs implemented. A further limitation of our study is that restrictions on uses of the multischool data set created for our study precluded identification of single-school data sets or cross-school comparisons, which may have been informative.

WBAs were available generally in numbers below what would be ideal for truly high-stakes decisions.^9,34,36,37^ Availability of WBAs may also be a surrogate for overall volume of assessment data typically collected on third-year clerkships.^43^ The validity and reliability of entrustment-focused assessments is limited^36,37^ and has been challenged on the grounds of subjectivity^46^ measurement–related issues,^8,47^ practical challenges,^7,45^ and default to presumption of readiness.^7^

## Conclusions

Our findings highlight substantial challenges in making prospective determinations about readiness of graduating medical students for indirect supervision in the Core EPAs framework at the scale of entire medical school classes.^7,8,33,45^ Results also suggest important gaps in readiness for a subset of Core EPAs (ie, EPAs 4, 8, 10, 11, and 13).^45^ Among next steps suggested by these results are improvement in curriculum and assessment specific to these EPAs at participating schools; further improvements in systems for programmatic assessment and data curation; and consideration of revision of what are considered Core EPAs on a specialty-specific basis for incoming interns.^45^

Identifying opportunities for direct observation, feedback, and faculty development related to assessment of these activities may help improve readiness. Nonetheless, there may be limited opportunity for meaningful participation and observation of these activities with advancement to performance of activities under indirect supervision prior to graduation for a variety of reasons involving culture and policy. It may be useful to implement a postmatch handoff process so that orientation boot camps, mentorship, and initial-year residency responsibilities may be tailored to specific educational needs of each incoming resident. Awareness of these gaps remains important for program directors to maintain patient safety and support medical school graduates’ educational needs upon starting residency. UME and GME educators need a shared mental model regarding required skills for incoming residents and what responsibility lies with medical schools vs specialty-specific organizations.^48^

## Notes

Supplement 1. **eTable.** Proportion of Students With Entrustment Determination of Ready for Indirect Supervision

Supplement 2. **Nonauthor Collaborators.** Core Entrustable Professional Activities for Entering Residency Pilot
