# Supplementary material for: Comparing Entrustment Decision-Making Outcomes of the Core Entrustable Professional Activities Pilot, 2019-2020
Source: JAMA Netw Open. 2022 Sep 26;5(9):e2233342. doi: 10.1001/jamanetworkopen.2022.33342 (PMC9513644; doi:10.1001/jamanetworkopen.2022.33342)
Supplement: Supplement 1. — eTable. Proportion of Students With Entrustment Determination of Ready for Indirect Supervision [file jamanetwopen-e2233342-s001.pdf]

Supplemental Online Content

Brown DR, Moeller JJ, Grbic D, et al; Core Entrustable Professional Activities for Entering Residency Pilot. Comparing entrustment decision-making outcomes of the core entrustable professional activities pilot, 2019-2020. *JAMA Netw Open*. 2022;5(9):e2233342. doi:10.1001/jamanetworkopen.2022.33342

eTable. Proportion of Students With Entrustment Determination of Ready for Indirect Supervision

This supplemental material has been provided by the authors to give readers additional information about their work.

© 2022 Brown DR et al. *JAMA Network Open.*

eTable. Proportion of Students With Entrustment Determination of Ready for Indirect Supervision[a]

EPA	2019: Total students, No. (%)	2020: Total students, No. (%)	Percentage point difference[b]	95% CI for difference	2-sided p-value
1	184/291 (63.2)	334/377 (88.6)	25.4	19.0 to 31.8	<.001
2	41/70 (58.6)	59/77 (76.6)	18.1	3.1 to 33.0	.02
3	22/69 (31.9)	58/77 (75.3)	43.4	28.8 to 58.1	<.001
4	9/79 (11.4)	6/74 (8.1)	-3.3	-12.7 to 6.1	.49
5	131/182 (72.0)	152/214 (71.0)	-0.9	-9.9 to 8.0	.83
6	210/286 (73.4)	315/333 (94.6)	21.2	15.5 to 26.8	<.001
7	164/193 (85.0)	144/195 (73.9)	-11.1	-19.1 to -3.2	.007
8	9/93 (9.7)	12/99 (12.1)	2.4	-6.4 to 11.2	.59
9	120/180 (66.7)	116/180 (64.4)	-2.2	-12.0 to 7.6	.66
10	2/72 (2.8)	3/72 (4.2)	1.4	-4.6 to 7.4	.65
11	1/62 (1.6)	0/66 (0.0)	-1.6	NA	NA
12	104/129 (80.6)	141/142 (99.3)	18.7	11.7 to 25.6	<.001
13	0/25 (0.0)	0/104 (0.0)	0.0	NA	NA

Abbreviations: CI, confidence interval; EPA, entrustable professional activity.

[a]Determination of “ready for indirect supervision” vs either “not progressing towards readiness” or “progressing towards readiness” determinations.

[b]Percentage point difference value may differ slightly from the subtraction of the 2019 percentage value from the 2020 percentage value due to rounding.

© 2022 Brown DR et al. *JAMA Network Open.*
