# Supplementary material for: Comparing Entrustment Decision-Making Outcomes of the Core Entrustable Professional Activities Pilot, 2019-2020
Source: JAMA Netw Open. 2022 Sep 26;5(9):e2233342. doi: 10.1001/jamanetworkopen.2022.33342 (PMC9513644; doi:10.1001/jamanetworkopen.2022.33342)
Supplement: Supplement 2. — Nonauthor Collaborators. Core Entrustable Professional Activities for Entering Residency Pilot [file jamanetwopen-e2233342-s002.pdf]

*Group Name(s): Core Entrustable Professional Activities for Entering Residency Pilot							
***First Name and Middle Initial(s)**	***Last Name**	***Suffix (eg, Jr, III)**	Academic Degrees	Institution	Location (city, state/province, country)	Role or Contribution, eg, chair, principal investigator	Group (if more than 1 Group listed in the byline) and/or Subgroup (eg, Steering Committee)
Jonathan	Amiel		MD	Columbia University Vagelos College	New York City, NY	Associate Project Lead, Steering Committee, Columbia Team Lead	Author
Beth	Barron		MD	Columbia University Vagelos College	New York City, NY	Curriculum and Assessment, Columbia Core Team	
Marina	Catallozzi		MD MSCE	Columbia University Vagelos College	New York City, NY	Faculty Development, Columbia Core Team	
Vivian	Obeso		MD	Florida International University (FIU)	Miami, FL	Steering Committee, FIU Team Lead, Curriculum and Assessment	Author
Jefry	Biehler		MD	Florida International University Herb	Miami, FL	Faculty Development, FIU Core Team	
David R	Brown		MD	Florida International University Herb	Miami, FL	Entrustment, FIU Core Team	Author
Mark	Hormann		MD	McGovern Medical School at the Uni	Houston, TX	UTHouston Team Lead, Steering Committee, Entrustment	Author
Sasha	Adams		MD	McGovern Medical School at the Uni	Houston, TX	UTHouston Core Team	
Allison R	Ownby		PhD	McGovern Medical School at the Uni	Houston, TX	Faculty Development, UTHouston Core Team	
Jennifer	Swails		MD	McGovern Medical School at the Uni	Houston, TX	UTHouston Core Team	

***First Name and Middle Initial(s)**	***Last Name**	***Suffix (eg, Jr, III)**	Academic Degrees	Institution	Location (city, state/province, country)	Role or Contribution, eg, chair, principal investigator	Group (if more than 1 Group listed in the byline) and/or Subgroup (eg, Steering Committee)
Dianne	Wagner		MD	Michigan State University (MSU) Coll	East Lansing, Michigan	MSU Team Lead, Steering Committee, Entrustment	
Matthew	Emery		MD	Michigan State University College of	East Lansing, Michigan	Curriculum and Assessment, MSU Core Team	
Aron	Sousa		MD	Michigan State University College of	East Lansing, Michigan	MSU Core Team	
Angela	Thompson-Busch		MD	Michigan State University College of	East Lansing, Michigan	Faculty Development, MSU Core Team	
Patrick M.	Cocks		MD	New York University (NYU) Grossman	New York City, NY	NYU Team Lead, Steering Committee, Entrustment	
Colleen C.	Gillespie		MD	NYU Grossman School of Medicine	New York City, NY	Curriculum and Assessment, NYU Core Team	
Melvin	Rosenfeld		MD	NYU Grossman School of Medicine	New York City, NY	Entrustment, NYU Core Team	
Linda	Tewksbury		MD	NYU Grossman School of Medicine	New York City, NY	Faculty Development, NYU Core Team	
George	Mejicano		MD MS	Oregon Health & Science University (	Portland, OR	OHSU Team Lead, Steering Committee	
Tracy	Bumsted		MD	Oregon Health & Science University S	Portland, OR	OHSU Core Team	
Carrie A.	Phillipi		MD PhD	Oregon Health & Science University S	Portland, OR	Curriculum and Assessment, OHSU Core Team	
Jamie	Warren		MD	Oregon Health & Science University S	Portland, OR	Entrustment, OHSU Core Team	

***First Name and Middle Initial(s)**	***Last Name**	***Suffix (eg, Jr, III)**	Academic Degrees	Institution	Location (city, state/province, country)	Role or Contribution, eg, chair, principal investigator	Group (if more than 1 Group listed in the byline) and/or Subgroup (eg, Steering Committee)
Sandra	Yingling		PhD	University of Illinois College of Medic	Chicago, IL	UICOM Team Lead, Steering Committee, Entrustment	
Meenakshy	Aiyer		MD	University of Illinois College of Medic	Chicago, IL	Curriculum and Assessment, UICOM Core Team	
Janet	Jokela		MD	University of Illinois College of Medic	Chicago, IL	Faculty Development, UICOM Core Team	
Asra R.	Khan		MD	University of Illinois College of Medic	Chicago, IL	UICOM Core Team	
William B.	Cutrer		MD MEd	Vanderbilt University School of Medi	Nashville, TN	Vanderbilt Team Lead, Steering Committee	Author
Cody	Chastain		MD	Vanderbilt University School of Medi	Nashville, TN	Vanderbilt Core Team	
Kendra	Parekh		MD MHPE	Vanderbilt University School of Medi	Nashville, TN	Vanderbilt Core Team	
Eduard	Vasilevskis		MD	Vanderbilt University School of Medi	Nashville, TN	Curriculum and Assessment, Vanderbilt Core Team	
Michael S.	Ryan		MD MEHP	Virginia Commonwealth University (V	Richmond, VA	VCU Team Lead, Steering Committee	
Diane M.	Biskobing		MD	Virginia Commonwealth University Sc	Richmond, VA	Entrustment, VCU Core Team	
Nicole	Deiorio		MD	Virginia Commonwealth University Sc	Richmond, VA	VCU Core Team	
Gregory	Trimble		MD	Virginia Commonwealth University Sc	Richmond, VA	VCU Core Team	
Michael	Green		MD	Yale School of Medicine	New Haven, CT	Yale Team Lead, Steering Committee	
Katherine	Gielissen		MD	Yale School of Medicine	New Haven, CT	Faculty Development, Yale Core Team	
Jeremy J.	Moeller		MD	Yale School of Medicine	New Haven, CT	Entrustment, Yale Core Team	Author

***First Name and Middle Initial(s)**	***Last Name**	***Suffix (eg, Jr, III)**	Academic Degrees	Institution	Location (city, state/province, country)	Role or Contribution, eg, chair, principal investigator	Group (if more than 1 Group listed in the byline) and/or Subgroup (eg, Steering Committee)
Barry	Wu		MD	Yale School of Medicine	New Haven, CT	Yale Core Team	
Alison J.	Whelan		MD	Association of American Medical Colleges (AAMC)	Washington, DC	Director of the Core EPAs Pilot	
